# Effect of isoniazid preventive therapy on tuberculosis incidence and its predictors among adult patients enrolled in antiretroviral therapy at public health facilities in Ambo Town, Oromia, Ethiopia: a retrospective cohort study

**DOI:** 10.1186/s12879-026-13569-z

**Published:** 2026-05-13

**Authors:** Milkesa Tesfaye, Tolessa Beyene, Meseret Ifa, Seifadin Ahmed, Dachasa Dabalo

**Affiliations:** 1 Communicable Disease Prevention and Control Office, Ambo Town Health Office, Ambo, Ethiopia; 2 College of Health Science and Referral Hospital, Ambo University, Ambo, Ethiopia; 3 College of Medicine and Health Sciences, Arsi University, Asella, Ethiopia; 4 Ethiopian Health Insurance Service, Ministry of Health, Addis Ababa, Ethiopia

**Keywords:** Isoniazid preventive therapy, Tuberculosis incidence, Ambo, Ethiopia

## Abstract

**Background:**

Tuberculosis is one of the major causes of illness and death among people living with the human immunodeficiency virus. The World Health Organization has recommended isoniazid preventive therapy for those patients to decrease tuberculosis-related infection and death.

**Objective:**

To assess the effect of isoniazid preventive therapy on tuberculosis incidence and its predictors among adult patients enrolled in antiretroviral therapy at public health facilities in Ambo Town, Ethiopia, between 2016 and 2021.

**Methods:**

An institution-based retrospective cohort study with a six-year follow-up from January 2016 to June 2021 was conducted from October 15, 2021, to November 10, 2021, among 771 (386 isoniazid-exposed and 385 non-isoniazid-exposed) adults on antiretroviral therapy at health institutions in Ambo Town. A simple random sampling technique was used to select patient records. Data were collected using a checklist and recorded on an electronic tool called ‘Kobo-Collect’ and exported to SPSS version 26.0 for further statistical analysis. Kaplan-Meier survival plots and the log-rank test were used to compare the crude effect in both the exposed and non-exposed groups on disease-free survival probabilities. Bivariable analysis was used to select candidates at a p-value < 0.25 and then entered multivariable Cox regression analysis to declare statistical significance at a p-value < 0.05 with the respective AHR.

**Results:**

In this study, isoniazid preventive therapy has a 90.7% reduction effect on tuberculosis incidence (AHR = 0.093, CI = 0.029–0.31). The incidence of tuberculosis among the isoniazid-treated group was 0.2 per 100 person-years and 2.2 per 100 person-years in the non-isoniazid group. Regarding predictors, those who did not receive isoniazid (AHR: 8.9; 95% CI: 2.52–31.61), were at WHO stage 3 (AHR: 15.5; 95% CI: 6.55–30.47), had a CD4 count < 100 cells/µl (AHR: 4.33 (1.35–13.88)), had a body mass index < 18.5 kg/m² (AHR: 2.86, 95% CI = 1.59–15.16), and had no previous tuberculosis treatment (AHR: 95% CI: 18.0 2.18–48.57) were significant predictors of incidence of tuberculosis infection.

**Conclusion:**

This study found a relatively lower tuberculosis incidence, though several significant predictors were observed in those with advanced WHO stage, CD4 count < 100 cells/µL, no prior tuberculosis treatment, and low baseline body mass index, underscoring the importance of isoniazid preventive therapy, which should be prioritized with integrated clinical, nutritional, and preventive interventions to reduce tuberculosis risk.

**Clinical trial number:**

Not applicable.

## Introduction

Globally, human immunodeficiency virus and acquired immunodeficiency syndrome (HIV/AIDS) remain the most significant global public health issue, with 39.9 million people living with the virus, 1.3 million newly infected, and 630,000 deaths from AIDS-related illnesses by 2023 [1]. Tuberculosis is a major cause of death for people with the human immunodeficiency virus (HIV), with active TB being higher in PLHIV patients and accounting for one-third of AIDS-related deaths worldwide. Globally in 2023, there were an estimated 161,000 deaths among people with HIV, in which TB was a contributing cause, while 1.09 million deaths occurred among HIV-negative people [2]. People living with human immunodeficiency virus (PLHIV) have a higher risk of developing active TB (15–22 times) than those without HIV. Globally, about 8.2% of the 10 million incident TB cases occurred among people living with HIV [3].

The World Health Organization (WHO) recommends enhanced TB case-finding, isoniazid preventive therapy, and infection control [4]. WHO and UNAIDS collaborate to recommend isoniazid preventive therapy (IPT) to decrease the incidence of tuberculosis (TB) among HIV-infected patients; IPT is effective preventive intervention in preventing active tuberculosis in HIV-infected patients, but its effectiveness in routine clinical systems is still lacking [5–7]. Despite antiretroviral therapy, PLHIV are three times more likely to die during treatment. Early initiation of ART, high screening standards, and extended preventive therapy are crucial interventions to ensure timely treatment for TB disease or infection in HIV-infected individuals [3, 8].

Ethiopia ranks 7th among high-burden countries with TB/HIV co-infection, with TB being the third leading cause of morbidity and mortality among PLHIV [7, 9, 10]. In 2016, an estimated 16,000 PLHIV in Ethiopia developed tuberculosis, with 54% receiving TB/HIV co-treatment [8, 11–14]. In Ethiopia, providing IPT reduces TB and death by 62% and 26%, respectively [15]. Ethiopian national guidelines for comprehensive HIV prevention, care, and treatment include eligibility for children over 12 months of age and people living with HIV who are screened negative for TB. Ethiopia, where IPT provision began in 2007, has a late initiation of IPT; Clients are provided IPT for six months, following the guidelines [8, 16]. Despite the provision of IPT, further investigation is needed to evaluate its advantages. IPT use may decrease TB incidence among PLHIV, but non-isoniazid preventive therapy users have a higher prevalence of TB. Factors affecting TB development include isoniazid use, co-trimoxazole preventive therapy, and ART [7, 14, 17]. The IPT use for HIV-infected individuals in Ethiopia are being challenged due to drug resistance, lack of IPT supply, poor healthcare provider commitment, and patients’ unwillingness to accept treatment [18, 19].

Even with the national IPT scale-up, evidence on its impact in routine clinical settings in Ethiopia remains limited. The effect of IPT on TB incidence and its predictors among HIV-infected individuals who were on IPT and never on IPT is not well studied in this area. So, the main aim of the study is to assess the effect of isoniazid preventive therapy on tuberculosis incidence and its predictors among adult patients enrolled on ART in Ambo town, Oromia regional state, Ethiopia; to highlight the gaps in providing IPT by illustrating the effect of IPT on TB incidence among PLHIV; and to support program managers in revising current strategies. Further showing the different predictors of TB incidence among PLHIV, which calls for improved strategies to reduce the incidence of tuberculosis among PLHIV, offers a chance for healthcare providers to minimize TB incidence and also serves as a baseline for future research.

## Methods and materials

### Study area and period

The study was conducted at public health facilities in Ambo town from October 15, 2021, to November 10, 2021, in the West Shewa Zone, Oromia regional state, Ethiopia, which is located 114 km west of Addis Ababa, the capital city of Ethiopia. Public health facilities within the city that had ART clinics were Ambo University Referral Hospital, Ambo General Hospital, Ambo Health Center, and Awaro Health Center [20].

### Study design

A facility-based retrospective cohort study, with a six-year follow-up (from January 2016 to June 2021), using secondary data from clinical charts of adult HIV patients receiving ART at four health facilities in Ambo town, was conducted.

### Population

#### Source population

All adult people living with HIV (PLHIV) who were enrolled in antiretroviral therapy (ART) at public health facilities in Ambo Town, Oromia Region, Ethiopia. The exposed group are adult patients who have taken IPT and enrolled in HIV care and treatment. The source population for the non-exposed group are those who had never taken IPT at Ambo town public health facilities.

#### Study population

All adult PLHIV who were enrolled in ART at public health facilities in Ambo Town during the study period and whose medical records were eligible for review (with documented IPT exposure status and TB outcome).

### Eligibility criteria

#### Inclusion Criteria

Adult patients enrolled in ART, aged 15 and above, who were free of active TB at the start of ART treatment and who had completed six months of isoniazid therapy, were considered eligible candidates and are included in the exposed group. Patients who had completed six months of IPT were eligible for the exposed cohort, while patients who had never initiated IPT were considered the unexposed group.

#### Exclusion Criteria

Incomplete patient charts or data on IPT completion or enrolment were excluded.

### Sample size determination

An independent cohort study determined the required sample size using Epi Info Version 7.1. It was assumed that isoniazid-complete subjects were exposed throughout the follow-up and untreated groups were unexposed. The incidence of TB among IPT-treated groups was 5.5% and 10.5% for non-IPT groups, taken from a previous similar study conducted in Nekemte town, Oromia, Ethiopia [12]. A power of 80% and a 5% margin of error, with an exposed-to-unexposed ratio of 1:1, is considered. Therefore, the final sample size was 501 for the unexposed and 501 for the exposed group. Since the total source population was less than 10,000 (3,213), a correction formula was used, and the final sample size was 771 (exposed group = 386 and unexposed group = 385).

### Sampling technique and procedure

**Fig. 1:**
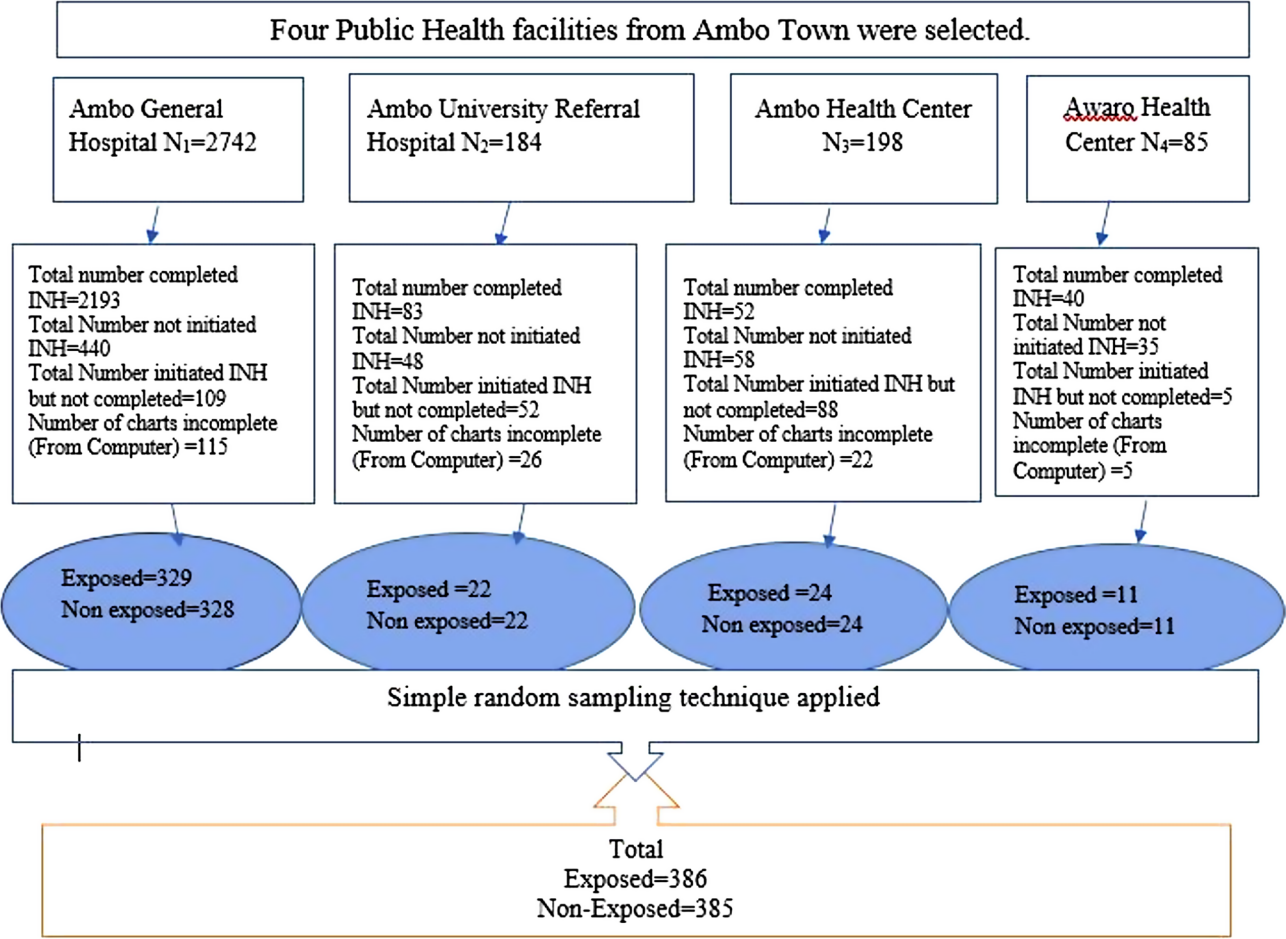
Figurative presentation of the sampling technique for the study on effect of isoniazid on TB incidence and its predictors among adult patients in Ambo Town public health facilities, Oromia Ethiopia, 2021

Based on the final sample size estimated for this study, proportional allocation was applied across all public health facilities in the town using the formula (**n**_**1**_ **= N**_**1**_**/N**_**T**_**) × n t**, where **n**_**1**_ = required sample size from each health facility, N_1_ = total number of source population at each health facility, **N**_**t**_ = total number of all adult PLHIV who were enrolled in antiretroviral therapy from all public health facilities and **n**_**t**_ = final sample size estimated for this study. So, for Ambo University Referral Hospital, which had 184 clients, **n ₁** = (184/3213) × 771 **= 44.** From those clients, 83 were eligible (IPT completed and age above 15), and 48 were eligible for the non-IPT group. For Ambo General Hospital, 2742 clients, **n ₂ =** 2742/3213 × 771 = **658.** From those, 2193 clients were suitable for the IPT group, and 440 clients were eligible for the non-IPT group. Ambo Health Center had 198 clients; **n ₃** = (198/3213) × 771 **= 48.** Of those, 52 clients were eligible, and 58 were suitable for the non-IPT group. Awaro Health Center had 85 clients, **n ₄ =** (85/3213) × 771 **= 20**,** and** from these, 40 and 35 clients were eligible for the non-IPT group, respectively. The relevant client records that met the requirements for inclusion in both cohort groups were listed. The exposed group was screened using the IPT registration logbook, and the unexposed group was screened using the ART registration logbook. Additionally, an electronic database was utilized to compile a list of clients on IPT and non-IPT to cross-check the logbook. The simple random sampling technique was used to select the representative sample from the smart care ART computer-based data of all public health facilities in the town, which has a list of unique ART numbers between 2016 and 2021. The final study population was selected based on their unique ART number until the predetermined sample size was attained for each cohort group (***as seen in*** Fig. 1*** below***).

### Study variables

#### Dependent variable

*Incidence of TB*.

### Independent variables

IPT receipt: IPT or no IPT; Sociodemographic variables: age, sex, educational status, occupation, and residence area. Clinical variables: WHO stage of HIV/AIDS, other opportunistic infections (OIs), CPT, BMI; Immunological Variable: CD4 count.

### Operational definitions

#### TB incidence

Occurrence of new TB cases during the follow-up period. This was ascertained per the national guideline, where routine screening during patient visits to health institutions will be done. If screened positive for the clinical screening, GeneXpert confirmation will be done, and the result will be stated on the patient’s follow-up form [21, 22].

#### IPT completed

Pre-ART or ART individuals taking isoniazid (INH) prophylaxis for six months to sterilize latent TB infection [19].

#### Adherence for ART

Good: ≥95% or ≤ 3 doses missed per month. Fair: 85–94% or 4–8 doses missed per month. Poor: <85% or ≥ 9 doses missed per month [16].

### Data collection tool

The data were collected using an English version of a data extraction form containing socio-demographic, clinical, and immunological factors. The form was developed by reviewing related literature [12, 19, 22].

### Data collection procedure

Data were collected by four data collectors (healthcare professionals who were assigned to ART provision) and one supervisor (public health expert) who were recruited. Data collectors identified and selected eligible records, then the extraction form was used to extract data. After identifying patients who completed IPT from 2016 to 2021, each individual was followed until either an active TB diagnosis (bacteriological or clinical decision) or censoring from their corresponding charts. Participants were censored at treatment completion, loss to follow-up, death, transfer, or end of study period. Those patients who were lost to follow-up, died, or transferred out of the study area during the cohort period were considered as censored data.

### Data quality assurance

The data quality was ensured using a well-structured data extraction form. There was a one-day training for the four data collectors and one supervisor regarding the data extraction form and how to select records/charts and extract data from them. Also, the assigned supervisor checked all the extracted data forms for completion and consistency on a daily basis.

### Data processing and analysis

Data were collected using the Kobo-Collect tool and exported to SPSS version 26 for cleaning and consistency checks. Then data was exported to Stata 14 software for further analysis. Each of the predictors was screened at a p-value ≤ 0.25 in the bivariable Cox regression analysis to fit into the multivariable Cox proportional hazard regression model. The assumptions for the Cox proportional hazard model were checked using the global test and the Schoenfeld residual test; no predictors violated the assumptions of the Cox proportional hazard model. Kaplan-Meier survival plots were used to estimate TB-free survival probabilities in exposed and non-exposed groups and compared using the log-rank test. Finally, the adjusted hazard ratio with its corresponding 95% confidence interval at *p* < 0.05 was identified as a significant predictor.

## Result

### Socio-demographic characteristics

A total cohort of 771 HIV patients on ART, including 386 IPT users and 385 non-IPT users, were followed for a median duration of 51 months. Four hundred sixty-three (60.1%) of the patients were female. Also, 531 (68.9%) lived in an urban area, while the rest were from rural areas. The mean baseline age of both cohorts was 39 ± 11.464 years; 464 (60.2%) were married at baseline, and 102 (13.2%) were single. The main religious group was Orthodox, containing 479 (62.1%), and 164 (21.3%) of the cohorts were educated up to the secondary school level (Table 1).

**Table 1:** Baseline socio-demographic characteristics of adult patients at the ART clinic of Ambo Town Public Health Facilities followed from January 2016 to June 2021, Ethiopia. (*n* = 771)

Variables	Category	Cohort Group		Total N (%)
		**On IPT n (%)**	**Non-IPT n (%)**	
Age Group	15–30	111 (28.8)	59 (15.3)	170 (22)
	31–45	189 (49)	188 (48.8)	377 (48.9)
	46–60	80 (20.7)	102 (26.5)	182 (23.61)
	> 61	6 (1.60)	36 (9.4)	42 (5.4)
Sex	Female	238 (61.7)	225 (58.4)	463 (60.1)
	Male	148 (38.4)	160 (41.5)	308 (39.9)
Residence area	Rural	140 (36.3)	100 (26)	240 (31.1)
	Urban	246 (63.7)	285 (74)	531 (68.9)
Marital Status	Single	55 (14.2)	47 (12.2)	102 (13.2)
	Married	226 (58.5)	238 (61.8)	464 (60.2)
	Separated	9 (2.30)	31 (8.1)	40 (5.2)
	Widowed	44 (11.4)	41 (10.6)	87 (11.0)
	Divorced	52 (13.5)	28 (7.3)	80 (10.4)
Educational Status	No Formal Education	122 (31.6)	94 (24.4)	216 (28)
	Primary	153 (39.9)	158 (41)	312 (40.5)
	Secondary	69 (17.9)	95 (24.4)	164 (21.3)
	Tertiary	41 (10.6)	38 (9.9)	79 (10.2)
Religion	Orthodox	228 (59.1)	251 (65.2)	479 (62.1)
	Protestant	144 (37.3)	121 (31.4)	265 (34.4)
	Muslim	3 (0.80)	4 (1.0)	7 (0.9)
	Others*	11 (2.80)	9 (2.3)	20 (2.6)

Footnotes: IPT: - Isoniazid preventive therapy

### Baseline clinical characteristics

This study showed that 40 (5.2%) of the entire cohort developed TB cases. The median CD4 count at baseline for the cohort was 348 (IQR 201–400). In this study, 723 (93.8%) of the cohort had working functional status at baseline to perform their daily duties. The majority (85.6%) of the entire cohort were categorized as WHO clinical stage I at the start of follow-up. The result showed that 69.6% of the study subjects had a BMI ≥ 18.5 at enrollment. Four hundred fifty-four (58.9%) of the patients were on co-trimoxazole preventive therapy, and 66 (8.6%) of the whole cohort had a history of previous TB treatment (Table 2).

**Table 2:** Baseline clinical and follow-up outcome characteristics of adult patients at ART of Ambo Town public health facilities followed from January 2016 to June 2021

Variables	Category	Cohort Group		Total N (%)
		On IPT n (%)	Non-IPT n (%)	
WHO Stage	Stage 1	352 (91.2)	308 (80.0)	660 (85.6)
	Stage 2	13 (3.4)	22 (5.7)	35 (4.5)
	Stage 3	20 (5.2)	49 (12.7)	69 (8.9)
	Stage 4	1 (0.30)	6 (1.60)	7 (0.90)
Previous-TB Treatment	Yes	15 (3.9)	51 (13.2)	66 (8.6)
	No	371 (96.1)	334 (86.8)	705 (91.4)
Functional Status	Working	375 (97.2)	348 (90.4)	723 (93.8)
	Ambulatory	10 (2.6)	30 (7.8)	40 (5.2)
	Bedridden	1 (0.3)	7 (1.8)	8 (1.0)
Baseline BMI	< 18.5	129 (33.4)	105 (27.3)	234 (30.4)
	≥ 18.5	257 (66.6)	280 (72.7)	537 (69.6)
CD4	< 100	36 (9.3)	32 (8.3)	68 (8.8)
	101–200	64 (16.6)	57 (14.8)	121 (15.7)
	201–350	112 (29)	134 (34.2)	246 (31.9)
	> 350	174 (45.0)	162 (42.1)	336 (43.6)
OIs other than TB	Yes	119 (30.8)	94 (24.4)	213 (27.6)
	No	267 (69.2)	291 (75.6)	558 (72.4)
CPT	Yes	213 (55.2)	241 (62.6)	454 (58.9)
	No	173 (44.8)	144 (37.4)	317 (41.1)
Outcome Status	TB	3 (0.80)	37 (9.6)	40 (5.2)
	No TB (censored)	383 (99.2)	348 (90.4)	731 (94.8)

Footnotes: CPT: cotrimoxazole preventive therapy; BMI: body mass index; CD4: Cluster differentiation 4; TB: tuberculosis; OIs: Opportunistic infections

### Incidence of tuberculosis

In this study, 771 participants were followed for 3092.88 person-years of observation. Forty (5.2%) participants developed TB while in follow-up, and 731 individuals were censored during follow-up, giving an overall incidence of TB during the follow-up period of 1.293 cases per 100 person-years. The TB incidence rate in the IPT group was 0.2 per 100 person-years and 2.2 per 100 person-years for the non-IPT group.

### Effect of isoniazid preventive therapy on tuberculosis incidence

**Fig. 2:**
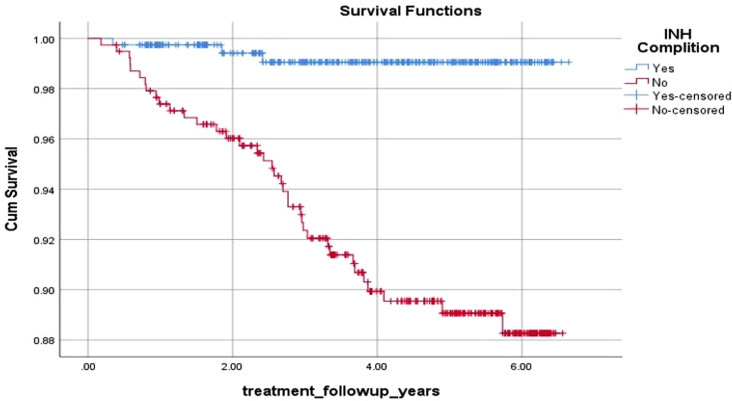
Kaplan-Meier survival estimates based on IPT among Adult HIV/AIDS patients on ART at Ambo town public health facilities, Oromia, Ethiopia, between 2016 and 2021

The risk of developing TB was reduced by 90.7% in the IPT group compared to the non-IPT group. Among the TB cases that occurred in the follow-up period, 21 cases occurred in females with an incidence of 0.68 per 100 person-years, and 36 (90%) of the TB cases were diagnosed in those between 15 and 60 years old in this age group. During the follow-up period, the highest TB rate was observed in patients with CD4 counts below 200 cells/µL at baseline, which is 25 (62.5%). Adult patients had significant differences in time to TB occurrence by having IPT. Those patients who took IPT had a longer survival time (TB-free) compared to those who did not (log-rank chi-square 24.462, p-value 0.001) (***as seen in*** Fig. 2***below).***

### Predictors of TB incidence among adult patients on ART

After fitting candidate variables into a multivariable Cox regression model, several baseline predictors were found to be significantly associated with TB incidence. Those include the WHO clinical stage of HIV/AIDS, baseline CD4 count, baseline BMI, and history of TB treatment. According to this study finding, the hazard of developing a tuberculosis infection among those with WHO clinical stage III is 15.5 times higher compared to those with other WHO clinical stages (I and II) (AHR = 15.5, 95% CI = 6.55–30.47). Furthermore, patients with a baseline CD4 count below 100 cells/µL had a 4.33 times higher hazard of developing a TB infection when compared with patients with a CD4 count greater than 350 cells/µL (AHR 4.33, 95% CI = 1.35 − 13.88). Additionally, patients with no history of TB treatment were 18 times more at risk of developing TB when compared to patients having a history of previous TB treatment (AHR 18, 95% CI = 2.18–48.57). Finally, the hazard of TB incidence among patients who have a baseline BMI less than 18 kg/m² is 2.86 times higher than those who have a baseline BMI greater than 18 kg/m² (AHR: 2.86, 95% CI = 1.59–15.16) (Table 3).

**Table 3:** Bi-variable and multivariable analysis on predictors of tuberculosis by using the Cox proportional hazard model of adults on ART at Ambo town public health facilities, followed from January 2016 to June 2021

Variables	Category	Disease outcome		CHR (95% CI)	AHR (95% CI)	*p*-value
		TB *n* (%)	No TB (*n* (%)			
Age Group	15–30	9 (22.5)	161 (22.0)	1	–	
	31–45	14 (35.0)	365(49.70)	0.69(0.31–1.59)		
	46–60	13(32.5)	169(23.10)	1.28 (0.55–2.98)		
	> 61	4 (10.0)	35 (5.20)	1.59 (0.49–5.19)		
Sex	Female	21(52.5)	442 (60.5)	0.69(0.37–1.27)	0.56(0.26–1.12)	0.13
	Male	19(47.5)	289 (39.5)	1	1	
Residence area	Rural	16 (40.0)	224 (30.6)	1	1	
	Urban	24 (60.0)	507 (69.4)	1.61 (0.85–3.03)	2.1 (0.109–4.42)	0.10
Marital Status	Single	8 (20.0)	94 (12.90)	1	1	
	Married	22 (55.0)	442 (60.50)	0.61(0.27–1.36)	1.12 (0.39–3.16)	0.84
	Separated	2 (5.0)	38 (5.20)	0.54(0.12–2.55)	0.48(0.18–2.61)	0.39
	Widowed	1 (2.5)	84 (11.50)	0.15(0.02–1.20)	0.44(0.15–4.14)	0.47
	Divorced	7 (17.5)	73 (10.0)	1.50(1.31–3.88)	1.2(0.01–1.91)	0.13
Educational Status	No Formal	9 (22.5)	207(28.3)	1.08 (0.29–4.0)	–	
	Primary	17(42.5)	295(40.4)	1.44(0.42–4.90)		
	Secondary	11(27.5)	153(20.9)	1.64(0.46–5.89)		
	Tertiary	3 (7.5)	76 (10.4)	1		
Occupational Status	Employee	3 (7.5)	107 (14.6)	1	1	
	Self–employee	2 (5)	54 (7.40)	1.45(0.24–8.67)	3.26(0.45–23.49)	0.24
	Merchant	6 (15)	72 (9.80)	2.88(0.72–11.54)	3.64(0.79–16.77)	0.90
	Farmer	11(27.5)	155 (21.2)	2.64(0.74–9.46)	1.71 (0.41–7.09)	0.46
	Housewife	8 (20.0)	116 (15.9)	2.31(0.61–8.71)	4.9 (0.13–21.34)	0.23
	Jobless	0	49 (6.70)	0	0.1(0.01–2.7)	0.97
	Daily Laborer	5 (12.5)	120 (16.4)	1.46 (0.35–6.1)	4.76(0.22–22.5)	0.40
	Others	5 (12.5)	58 (7.90)	2.8 (0.69–12.16)	0.31(0.04–2.15)	0.24
WHO Stage	Stage 1	14 (35)	646 (88.4)	1	1	1
	Stage 2	2 (5.0)	33 (4.50)	3.03 (0.69–13.32)	2.04 (0.44–9.49)	0.1
	Stage 3	24 (60.0)	45 (6.20)	21.41 (11.05–41.47)	15.5 (6.55–30.47)	0.001*
	Stage 4	0	7 (0.96)	0.01(0.001–47.46)	0.12 (0.1–10.2)	0.91
Previous TB treatment	Yes	1 (2.5)	65 (8.90)	1	1	
	No	39(97.5)	666 (91.1)	4.21 (0.58–30.54)	18.0 (2.18–48.57)	0.02*
Functional Status	Working	28(70.0)	695 (95.1)	1	1	
	Ambulatory	11(27.5)	29 (3.97)	7.39(3.68–14.86)	0.74(0.3–2.33)	0.59
	Bedridden	1 (2.5)	7 (0.96)	3.7(0.5–27.28)	0.79(0.67–9.4)	0.83
Baseline BMI	< 18.5 kg/m2	23(57.5)	211(28.9)	3.10 (1.69 − 5.69)	2.86 (1.59–15.16).	0.04*
	≥ 18.5	17(42.5)	520 (71.1)	1	1	
CD4	< 100	9 (22.5)	59 (8.07)	7.41(2.60–20.81)	4.33(1.35–13.88)	0.01*
	101–200	16 (40.0)	230 (31.5)	3.60(1.43–9.30)	1.47(0.05–4.03)	0.46
	201–350	9 (22.5)	112 (15.3)	4.17(1.48–11.7)	1.004(0.40–2.51)	0.99
	> 350	6 (15.0)	330 (45.1)	1	1	
Opportunistic Infection	Yes	29(72.5)	529 (72.4)	1.05(0.5–2.01)	–	
	No	11(27.5)	202 (27.6)	1		
CPT	Yes	35(87.5)	419(57.3)	1	1	
	No	5 (12.5)	312 (42.7)	0.21(0.08–0.54)	0.28(0.09 − 1.84)	0.12
Cohort Group	On IPT	3 (7.50)	383(52.4)	1	1	
	Non-IPT	37(92.5)	348(47.6)	12.315(3.31–34.89)	8.9(2.52–31.61)	0.001*

Footnote: ‘–’ indicates the predictor is not a candidate for multivariable. Cox regression analysis at p-value < 0.25; 1: reference categories; *: significant predictors in multi-variable Cox regression model at p-value < 0.05; CPT: cotrimoxazole preventive therapy; BMI: body mass index; CD4: cluster differentiation 4; TB: tuberculosis; IPT: isoniazid preventive therapy

### Effects of IPT on TB incidence

After adjusting for other predictors, the overall effect of IPT was found to reduce TB incidence by 90.7% (AHR = 0.093, CI = 0.029–0.31). Each independent variable was evaluated concerning IPT exposure, or IPT was adjusted for each of the predictors independently; none of the independent predictors modified the effect of IPT by more than 10. The multivariable effect of IPT on each independent variable’s subgroup was also examined using interaction analysis, but except for WHO stages (p-value = 0.001), BMI (p-value = 0.001), and CD4 count (p-value = 0.02), the p-value was statistically insignificant. Also, it did not show any variation effect of IPT on the other independent variables (Table 4).

**Table 4:** Effect of IPT on reduction of tuberculosis incidence among cohorts of adult patients on ART at Ambo town public health facilities followed from January 2016 to June 2021

IPT-adjusted covariates	CHR 95% CI	*p*-value	AHR (95% CI)	*p*-value	Reduction in TB (%)	*p*-value Interaction
IPT	0.093(0.03–0.31)	0.001*	0.09(0.025–0.33)	0.001	91.0	
IPT*CD4 Count			0.095(0.03–0.31)	0.001	90.5	0.02
IPT*WHO stages			0.162(0.049–0.54)	0.003	83.8	0.001
IPT*BMI			0.082(0.03–0.27)	0.001	91.8	0.001
IPT*Previous TB treatment			0.086(0.03–0.28)	0.001	91.4	0.08

Footnote: BMI: body mass index; CD4: cluster differentiation 4; TB: tuberculosis, WHO: World health organization; CHR: crude hazard ratio

## Discussion

In this research, individuals on ART had an overall incidence of TB of 1.29 per 100 person-years. This is less than previous studies conducted in Ethiopia and Tanzania, which found that individuals on ART had TB incidences of 3.57 and 2.7 per 100 person-years, respectively [13, 23]. According to this study, individuals not receiving IPT had a risk of TB incidence of 2.2 per 100 person-years, but those receiving IPT prophylaxis had a TB incidence of 0.2 per 100 person-years. This finding is in line with research carried out in Ethiopia and other nations with a high TB burden. Furthermore, in this study, IPT prophylaxis has been shown to be independently associated with a 90% lower hazard of TB incidence; this result is higher when compared with other studies conducted in different regions of Ethiopia and other countries with higher TB prevalence. The study conducted in Tanzania found a 48% reduction of tuberculosis incidence, which was solely accredited to IPT used for HIV-positive clients [23]. Similarly, another retrospective study in southern Ethiopia within a comparable study setting found that the combined effect of IPT and ART, when started simultaneously, demonstrated a 57% lower hazard of TB relative to ART alone [13]. The observed differences in IPT effect size between the current study and the previous one may be explained by variations in study design, follow-up duration, and baseline characteristics of the study populations. In particular, differences in the distribution of key clinical factors such as CD4 count, WHO clinical stage, and nutritional status may influence the baseline risk of TB and thereby affect the relative impact of IPT. Additionally, variations in adherence to IPT, timing and duration of IPT relative to ART, and differences in case ascertainment methods across studies may contribute to the observed discrepancies [24, 25]. Residual confounding inherent in retrospective cohort studies should also be considered. Therefore, the magnitude of the IPT effect observed in this study should be interpreted in light of these methodological and population differences rather than unmeasured facility-level factors.

Additionally, this study found several predictors of TB incidence among PLHIV, regardless of their IPT status. Baseline clinical factors such as the WHO clinical stage, history of previous TB treatment, a body mass index (BMI) below 18.5 kg/m², and a low CD4 count were among the main predictors for the occurrence of TB. Although TB can develop at any point of the HIV clinical course, comparing patients in WHO clinical stage III to those at stages I or II was associated with a 15.5-fold increase in TB incidence. The incidence of TB and advanced HIV stages have been linked, as revealed by other studies conducted in Ethiopia [12, 22].

In this study, individuals with no history of previous TB treatment had a significantly higher hazard of incident TB. Although this finding appears counterintuitive compared with some reports in the literature, it should be interpreted with caution. One possible explanation is that previous TB treatment may serve as a proxy for earlier diagnosis and closer clinical follow-up, which could facilitate earlier detection and management of subsequent TB risk. Additionally, individuals without prior TB treatment may represent a subgroup with undetected or delayed access to health services, potentially increasing vulnerability to progression from infection to active disease in high TB-burden settings. However, important variables such as TB contact history, household exposure, and environmental risk factors were not measured in this study, limiting the ability to fully explain this association. Therefore, this result should be considered hypothesis-generating, and further studies incorporating detailed exposure assessment are needed to clarify this relationship [26–28].

This study revealed a relationship between the incidence of TB and a CD4 count of less than 100 cells/µL, which was discovered to be a significant predictor of TB occurrence. In the current study, those with CD4 counts below 100 cells/µL had a 4.33-fold higher hazard of incident TB than individuals with CD4 counts over 350 cells/µL. Many studies with a different range of hazards have revealed similar results. This study finding was consistent with research conducted in Ethiopia, which revealed that patients with lower CD4 levels are at a two to three times higher risk [29–32]. This consistency between the current finding and other studies may be explained by immunological mechanisms due to the immune system. Impairment related to CD4 and T-cells is pivotal in orchestrating the immune response against infections, including TB infection, so a decline in CD4 count is known to impair cell-mediated immunity, which may increase susceptibility to TB. Another way to see the biological plausibility of CD4 count and TB infection is that they increase bacterial load and dissemination [33].

In addition, individuals with a BMI under 18.5 kg/m² had around a three-fold increased hazard of TB infection when compared with those who have a body mass index greater than 18.5 kg/m². This finding is consistent with research in northwest Ethiopia that revealed patients with a BMI of less than 18.5 at baseline [34]. Another prospective study from Tanzania found that lower BMI and falling BMI in HIV-positive patients were strong predictors of active TB [23]. The association between low BMI and a higher risk of TB incidence among HIV-infected individuals is biologically and epidemiologically plausible. Undernutrition is known to impair both innate and adaptive immunity, which may increase vulnerability to TB, which is essential for controlling Mycobacterium tuberculosis. HIV-infected patients with a BMI of less than 18.5 kg/m² are therefore more vulnerable to reactivation of latent TB or progression to active disease [35–39]. Even though patients’ baseline functionality status was not a statistically significant predictor in this study, the research conducted somewhere in Ethiopia [12, 22, 40], underscored that the risk of developing TB would be higher among bedridden than those ambulatory at baseline. This discrepancy might be occurred due to unmeasured residual confounding such socioeconomic status, ART adherence level, or unreported comorbidities might affect the finding of current study.

### Limitation and strength of the study

This study may be biased because it is a retrospective observational cohort study. The elimination of incomplete charts and reliance on accessible medical information may have led to selection bias. The extraction of exposure and outcome data from regular clinical records, which may be susceptible to misclassification, raises the possibility of information bias. Even while measurable confounders were adjusted for using multivariable Cox regression, residual confounding from unmeasured characteristics such as socioeconomic status, ART adherence level, or unreported comorbidities cannot be ruled out. Consequently, these intrinsic limits of observational data should be taken into account when interpreting results. Furthermore, due to the retrospective nature of this cohort study, all study participants’ charts were not included in the study analysis due to the incompleteness of some important variables like viral load, haemoglobin, contact history, environmental factors and ART drug regimen. Despite this limitation, the study has a long follow-up period (six years), which extended the observation period and allowed researchers to learn more about the long-term effects of chronic HIV care with IPT provision and potential predictors of TB incidence.

## Conclusion

Compared to most of the previous research, the incidence rate of TB is lower in this study, while several significant predictors of tuberculosis occurrence were observed regardless of IPT status. Advanced WHO stage (stage 3) strongly predicted TB incidence, reflecting the role of immunosuppression. Patients without prior TB treatment history were more likely to develop TB, possibly indicating undiagnosed or latent TB reactivation. Low baseline BMI (< 18.5 kg/m²) and severe immunosuppression (CD4 < 100) significantly higher risk of TB incidence. Thus, it highlights that strengthening IPT adherence may further reduce TB incidence. Additionally, these findings underline and highlight the importance of nutritional intervention and immune status; TB prevention efforts among adults on ART should be strengthened through integrated interventions, need to be emphasized for targeted screening, and should be prioritized for patients with predictive risk factors underscored in this study, and focused clinical follow-up should be for the patients with advanced HIV/AIDS stage and those with CD4 counts below 100 cells/µl, alongside nutritional support for underweight clients to enhance immunity. Moreover, ensuring consistent provision and adherence to isoniazid preventive therapy and improving screening for latent TB in patients without prior TB history and treatment are essential.

## Data Availability

The data that support the findings of this study are available from the corresponding author upon reasonable request.

## Abbreviations

AHR: Adjusted Hazard Ratio
AIDS: Acquired Immune Deficiency Syndrome
ART: Antiretroviral Therapy
BMI: Body Mass Index
CI: Confidence Interval
HAART: Highly Active Antiretroviral Therapy
HIV: Human Immunodeficiency Virus
ICF: Intensified Tuberculosis Case Finding
INH: Isoniazid
IPT: Isoniazid Preventive Therapy
IQR: Inter Quartile Range
IRR: Incidence Rate Ratio
MDR: Multidrug resistant
MTB: Mycobacterium Tuberculosis
OI: Opportunistic Infections
PLHIV: People Living with HIV
PY: Person-Years
SD: Standard Deviation
TB: Tuberculosis
UNAIDS: United Nations Program on HIV/AIDS
